# Effectiveness of Lomustine Combined With Bevacizumab in Glioblastoma: A Meta-Analysis

**DOI:** 10.3389/fneur.2020.603947

**Published:** 2021-01-20

**Authors:** Xing Ren, Di Ai, Tong Li, Lei Xia, Lingzhi Sun

**Affiliations:** ^1^College of Traditional Chinese Medicine, Shandong University of Traditional Chinese Medicine, Jinan, China; ^2^College of Health, Shandong University of Traditional Chinese Medicine, Jinan, China; ^3^The First Clinical Medical College, Shandong University of Traditional Chinese Medicine, Jinan, China; ^4^School of Energy and Power Engineering, Shandong University, Jinan, China; ^5^Department of Neurology, Affiliated Hospital of Shandong University of Traditional Chinese Medicine, Jinan, China

**Keywords:** glioblastoma, lomustine, bevacizumab, meta-analysis, dose

## Abstract

**Introduction:** Despite surgical and chemotherapeutical treatment options, the prognosis for glioblastoma (GBM) remains poor. Some studies have found that using lomustine plus bevacizumab to treat GBM can prolong overall survival (OS) and progression-free survival (PFS). The aim of this study was to explore the efficacy of the two drugs in combination treatment of GBM using a meta-analysis of the existing literature to help settle the ongoing debate.

**Materials and Methods:** PubMed, EMBASE, and the Cochrane Library were searched for the effectiveness of lomustine plus bevacizumab in GBM literature, updated on June 6, 2020. The main outcomes analyzed included PFS and OS; the effects of this drug combination on the 6-month PFS, which represents the percentage of patients who had PFS for 6 months, were also analyzed. All the data were pooled: OS and PFS with the mean difference (MD) and 6-month PFS with the risk ratio (RR). Because there were different control groups and dose groups, two subgroup analyses were run to ensure they were comparable. All statistical analyses were performed using the Review Manager Version 5.3 software.

**Results:** Six clinical trials were identified which included 1,095 patients (treatment group: 516; control group: 579). The group treated with lomustine and bevacizumab showed an improvement in OS (MD =1.37; 95% CI, 0.49–2.25; *p* = 0.002), PFS (MD = 0.23; 95% CI, 0.13–0.34; *p* < 0.00001), and 6-month PFS (RR = 2.29; 95% CI, 1.43–3.65; *p* = 0.0005). Two subgroup analyses of the main outcome, OS, show that the results of Control group A (*p* = 0.01) and Dose group 2 (*p* = 0.003) are significantly different from those of the other control or dose groups.

**Conclusion:** This study shows that lomustine and bevacizumab can effectively increase OS, PFS, and 6-month PFS in patients with GBM. The encouraging results of the lomustine and bevacizumab combination therapy for GBM should be studied in more clinical trials in the future.

## Introduction

Glioblastoma is the most aggressive glioma (WHO Grade IV) and is associated with a uniformly poor prognosis (1). The current standard treatment for glioblastoma (GBM) consists of a multimodality approach which includes maximal surgical resection and radiotherapy with concurrent temozolomide, followed by cycles of adjuvant chemotherapy (2). Despite multimodality treatments, recent clinical trials have reported a median survival of only 14–16 months with a 2-year survival rate of 26–33% (2, 3). There is therefore an urgent need to explore new therapeutic strategies to improve patient prognosis.

Glioblastoma multiforme is a highly vascularized tumor where the vascular endothelial growth factor (VEGF) pathway is up-regulated, and it has been hypothesized that GBM would respond well to antiangiogenic treatments (4). Bevacizumab (BEV) is an antibody against the vascular endothelial growth factor receptor (VEGF) and a common therapy used for colorectal, lung, breast, kidney, and ovarian cancers (5–7). In 2009, bevacizumab was approved by the Food and Drug Administration for use as a treatment of recurrent glioblastoma (8). Despite obvious radiographic responses and an observed increase in progression-free survival (PFS), some clinical studies which investigated BEV reported that treatment has not resulted in a durable overall survival (OS) benefit in either recurrent or newly diagnosed GBM (9–12).

In a phase III study, enzastaurin was compared with lomustine, an alkylating agent of the nitrosourea family that is widely used as a salvage treatment drug, and lomustine was found to be more effective in treating GBM, suggesting that nitrosourea plays an important role in the treatment of recurrent GBM (13). A previous meta-analysis (14) study explored the efficacy of bevacizumab plus lomustine treatment in progressive glioblastoma, but the studies included were so few that the results were controversial, and the correlation subgroup analysis was incomplete. Thus, a further statistical analysis is needed to increase the credibility of this treatment combination.

## Materials and Methods

This meta-analysis was performed according to the Cochrane Handbook for Systematic Reviews of Interventions and is presented based on the Preferred Reporting Items for Systematic Reviews and Meta-analyses guidelines. The protocol for this meta-analysis is available in PROSPERO (CRD42020190739).

### Inclusion Criteria

Studies that met the following criteria were included in the meta-analysis: Population: recurrent GBM adult patients (≥18 years old), with a Karnofsky Performance Status score ≥50 or a WHO Performance Status score between 0 and 2, were used. Intervention: lomustine plus bevacizumab; Comparison: monotherapy of bevacizumab, lomustine, or bevacizumab plus irinotecan. Outcome: the outcomes of interest were OS and PFS or 6-month PFS, and their corresponding 95% confidence intervals (CIs) were provided; Non-English language literature was excluded. In addition, when we found duplicated or overlapping data in multiple reports, we included the one with the most complete information.

### Search Strategy

Two investigators independently searched the electronic databases PubMed, EMBASE, and the Cochrane Library for relevant literature published up until June 2020. The search syntax included the following text words: “glioblastoma,” “bevacizumab,” “lomustine,” and “CCNU.” The detailed search strategy is available in the Supplementary Material.

### Data Extraction

All data were reviewed and separately computed by two independent investigators. The following information was extracted from each trial: median OS, median PFS, 6-month PFS, study design, control group measures, isocitrate dehydrogenase (IDH) status, promoter of O6-methylguanine-DNA-methyltransferase (MGMT) status, Karnofsky Performance Status or WHO Performance Status score, drug dose, and the number and age of the patients in the experimental and control arms.

### Quality Assessment

Two investigators separately rated the quality of the retrieved studies. We chose the risk-of-bias items recommended by The Cochrane Handbook for Systematic Reviews of Interventions for randomized controlled trials (RCTs). Items were evaluated in three categories: low risk of bias, unclear bias, and high risk of bias. In addition, we used the Methodological Index for Non-Randomized Studies (MINORS) for other clinically controlled trials.

### Statistical Analysis

The statistical analyses were performed using the Review Manager Version 5.3 software (RevMan; The Cochrane Collaboration, Oxford, UK). The end points of interest in the pooled analysis were OS, PFS, and 6-month PFS. Because the outcome index is a continuous variable, the mean difference (MD) was used as the effect index. Heterogeneity across studies was examined using the *I*^2^ statistic (15). Studies with an *I*^2^ of 25–50%, 50–75%, or >75% were considered to have low, moderate, or high heterogeneity, respectively (16). We used the random-effects model of statistical analysis and a value of *p* < 0.05 indicated statistical significance. In addition, we did a subgroup analysis based on dose and measures in the control groups in order to analyze the factors that influence disease response and a sensitivity analysis to find the sources of heterogeneity.

## Results

### Overview of the Literature Search

**Figure 1:**
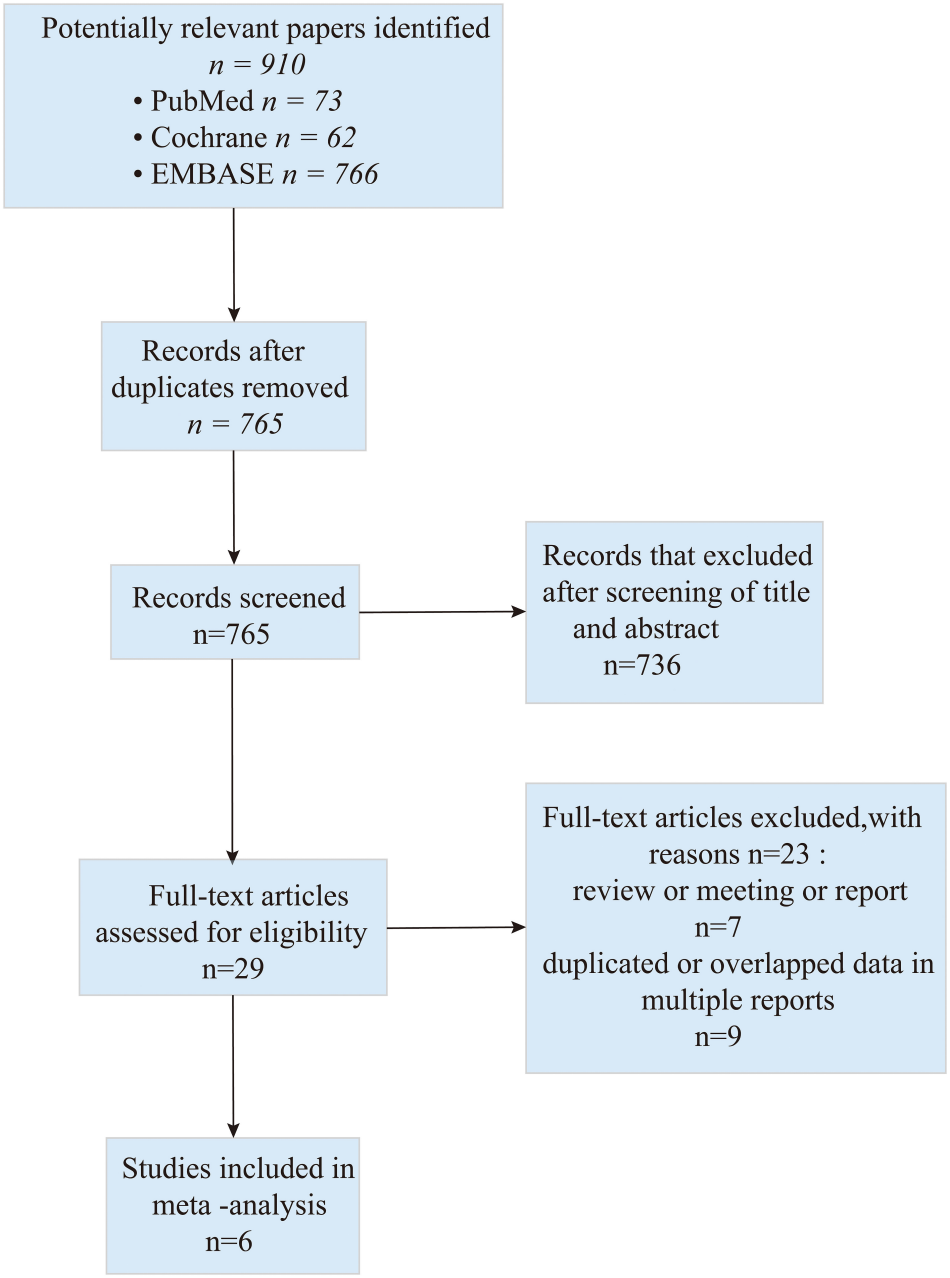
Flow diagram of study selection based on the PRISMA statement.

A total of 901 studies were retrieved initially for the evaluation. We did the initial screening based on the title and abstract, and 29 publications were chosen for further analysis. Eventually, six studies (one of the studies was split into two groups) (17–22) which addressed the combination of bevacizumab and lomustine in treating GBM were included in this study. The search process is described in Figure 1.

### Study Characteristics and Bias Risk Assessment Results

All included studies in this study were based on moderate to high-quality evidence. The primary characteristics of the six studies are detailed in Table 1. Of the six studies included, four were RCTs and two were non-RCTs. According to the type of study, we used The Cochrane Handbook for Systematic Reviews of Interventions and MINORS to assess the risk of bias, respectively. The results of the quality assessment results are shown in the Supplementary Tables 1, 2.

**Table 1:** Basic characteristics of patients in the included studies.

**Author**	**Year**	**Study design**	**Arms**	**Subjects**	**Age**	**Female%**	**IDH Mutated**	**MGMT Methylated**	**KPS**	**WHOPS**	**Outcome**
Heiland	2016	RS	Lom + Bev	18	50	50%	2/18	4/18	≥60	NR	Median OS Median PFS
			Bev	17	39.5	41%	3/17	6/17			
Weathers	2016	RCT	Lom + Bev	35	53.32	67%	NR	NR	≥60	NR	Median OS Median PFS 6-month PFS
			Bev	36	53.86	69%	NR	NR			
Brandes	2019	RCT	Lom + Bev	61	56	28%	NR	11/61	≥60	NR	Median OS Median PFS
			Lom + Placebo	62	58.5	27%	NR	12/62	≥50		
Taal	2014a	RCT	Lom + Bev	22	58	32%	2/20	10/21	NR	0–2	Median OS Median PFS 6-month PFS
			Bev	50	58	36%	1/39	18/42			
Taal	2014b	RCT	Lom + Bev	22	58	32%	2/20	9/20	NR	0–2	Median OS Median PFS 6-month
			Lom	46	56	43%	3/42	23/43			
Jakobsen	2018	PS	Lom + Bev	70	62	37.10%	NR	NR	NR	0–2	Median OS Median PFS
			Bev + Iri	219	56	33.30%	NR	NR			
Wick	2017	RCT	Lom + Bev	288	57.1	39.60%	NR	67/288	NR	0–2	Median OS Median PFS
			Lom	149	59.8	38.90%	NR	37,149			

*IDH, isocitrate dehydrogenase; MGMT,O6-methylguanine-DNA-methyltransferase;KPS, Karnofsky Performance Status; WHOPS, World Health Organization performance status; RS, retrospective study; RCT, randomized controlled trial; PS, prospective study; Lom, lomustine; Bev, bevacizumab; NR, not report*.

### Clinical Effect With the Combination of Bevacizumab and Lomustine in GBM

#### Pooled Analysis of OS

**Figure 2:**
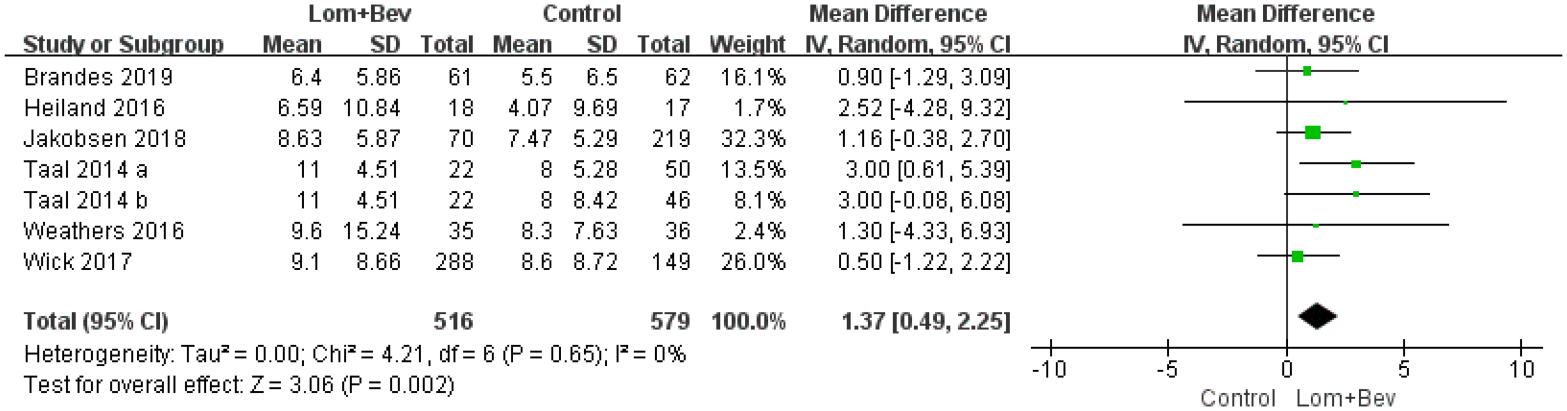
Forest plot of OS in glioblastoma between lomustine plus bevacizumab and control groups. OS, overall survival; Lom, lomustine; Bev, bevacizumab.

Pooling the OS data from the six studies (17–22) showed that bevacizumab combined with lomustine did prolong OS (MD =1.37; 95% CI, 0.49–2.25; *p* = 0.002) when compared to OS in the bevacizumab or lomustine monotherapy groups and the bevacizumab plus irinotecan group (Figure 2).

#### Pooled Analysis of PFS and Sensitivity Analysis

**Figure 3:**
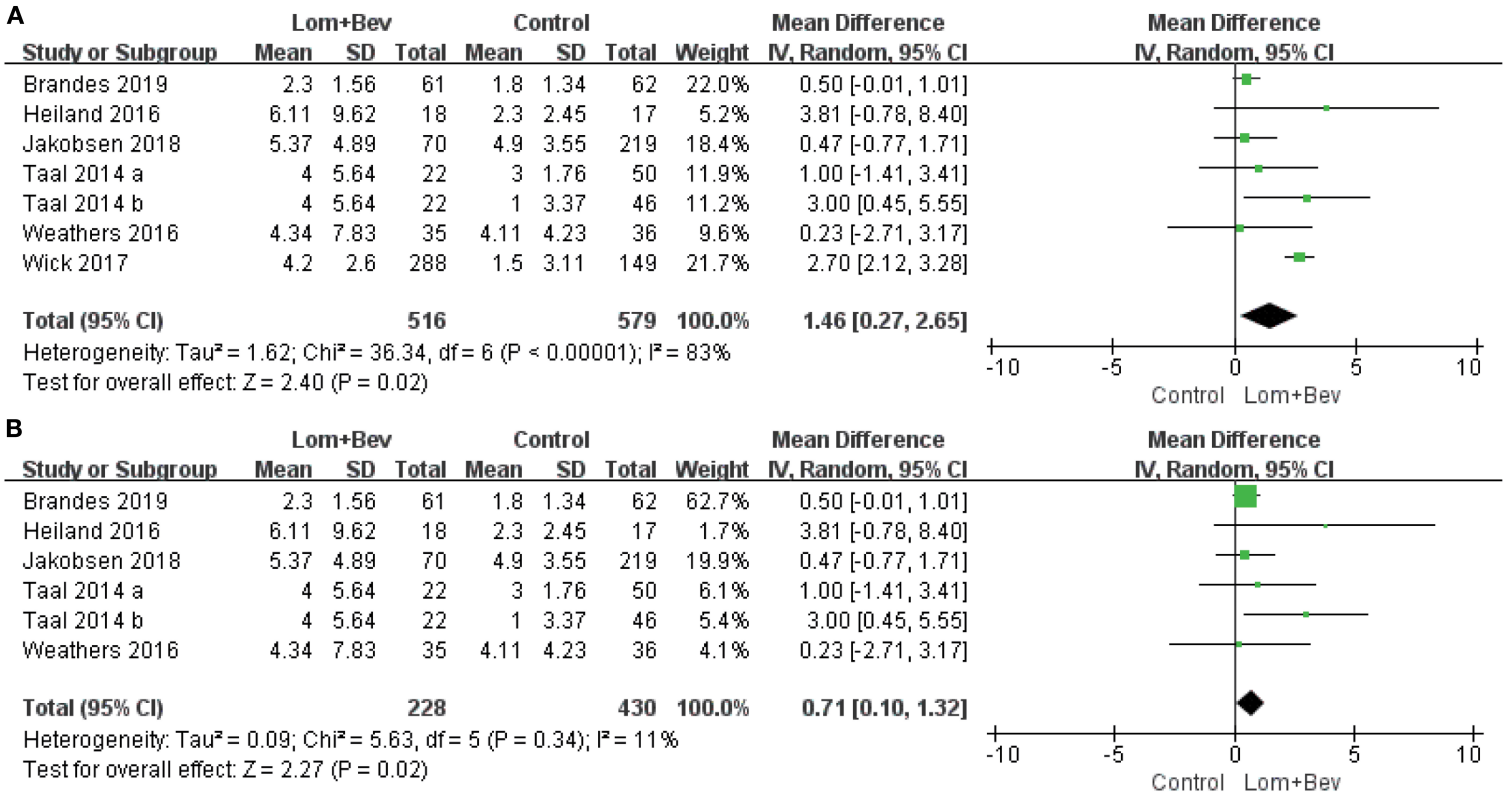
Forest plot of PFS in glioblastoma between lomustine plus bevacizumab and control groups. **(A)** All of the studies were included. **(B)** The study of Wick was removed. PFS, progression-free survival; Lom, lomustine; Bev, bevacizumab.

A random effects model was used to pool the PFS data (17–22). Despite the high degree of heterogeneity (*I*^2^ = 83%), the pooled data showed that the combination of bevacizumab and lomustine resulted in longer PFS (MD = 1.46; 95% CI, 0.27–2.65; *p* = 0.02) than in the control groups. We then looked for heterogeneous sources based on the sensitivity analysis. The results showed a significant decrease in heterogeneity (*I*^2^ = 11%) when the study of Wick (20) was removed and still had statistical significance (*p* = 0.02). This data indicates the results were robust (Figure 3).

#### Pooled Analysis of 6-Month PFS

**Figure 4:**
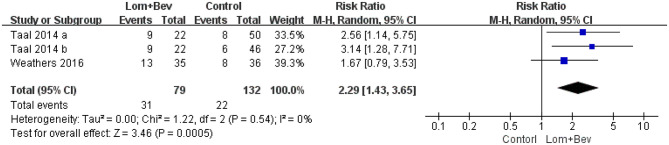
Forest plot of 6-month PFS in glioblastoma between lomustine plus bevacizumab and control groups. Six-month PFS, the percentage of patients who could progression-free survival for 6 months; Lom, lomustine; Bev, bevacizumab.

Pooling the 6-month PFS data from two of the studies (17, 19) showed that bevacizumab plus lomustine did improve the 6-month PFS (RR = 2.29; 95% CI, 1.43–3.65; *p* < 0.0005) compared with that in the bevacizumab or lomustine monotherapy groups (Figure 4).

#### Subgroup Analysis of OS

**Figure 5:**
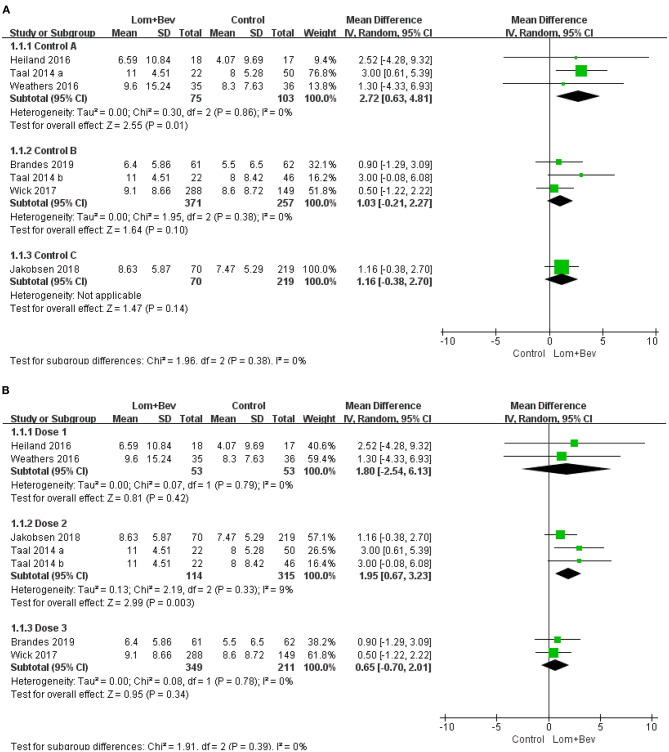
Subgroup analysis of OS in glioblastoma according to **(A)** different control groups, **(B)** different dose groups. Control group A, bevacizumab monotherapy; Control group B, lomustine monotherapy or lomustine plus placebo; Control group C, bevacizumab plus irinotecan; Dose1, Bev(5 mg/kg every 3 weeks) + Lom(90 mg/m^2^ every 6 weeks); Dose2, Bev (10 mg/kg every 2 weeks) + Lom (90 mg/m^2^ every 6 weeks); Dose3, Bev(10 mg/kg every 2 weeks) + Lom (90–200mg/m^2^ every 6 weeks).

First, we divided the patient data into Control group A (bevacizumab monotherapy; MD = 2.72; 95% CI, 0.63–4.81; *p* = 0.01), Control group B (lomustine monotherapy or lomustine plus placebo; MD = 1.03; 95% CI, −0.21 to 2.27; *p* = 0.10), and Control group C (bevacizumab plus irinotecan; MD = 1.16; 95% CI, −0.38 to 2.7; *p* = 0.14). Only Control group A showed statistical significance. Based on the different doses used in each study, we then divided the patients into Dose group 1 [Bev (5 mg/kg every 3 weeks) + Lom (90 mg/m^2^ every 6 weeks)]; MD = 1.80; 95% CI, −2.54 to 6.13; *p* = 0.42, Dose group 2 [Bev (10 mg/kg every 2 weeks) + Lom (90 mg/m^2^ every 6 weeks)]; MD = 1.95; 95% CI, 0.67−3.23; *p* = 0.003, and Dose group 3 [Bev (10 mg/kg every 2 weeks) + Lom (90–200 mg/m^2^ every 6 weeks)]; MD = 0.65; 95% CI, −0.70 to 2.01; *p* = 0.34. The results show that only the results of Dose group 2 had statistical significance. A forest plot of all subgroup analyses is shown in Figure 5.

## Discussion

Glioblastoma (GBM) is one of the most aggressive brain cancers in adults (2). Despite surgical treatment and chemotherapy options, the prognosis for patients remains poor (23). Bevacizumab, a monoclonal antibody against VEGF-A, used alone or in combination with cytotoxic drugs, showed interesting results in terms of radiographic response rates and PFS in initial phase 2 studies of GBM (11, 24). However, these studies lacked a non-bevacizumab control group and OS did not increase with the experimental group compared to the control group. Another study showed that early treatment with bevacizumab improved PFS, but not OS, again suggesting that the treatment with bevacizumab alone might not be sufficient in improving GBM patient prognosis (25). Thus, we looked for another drug to combine with bevacizumab to improve OS and chose lomustine, a DNA alkylating agent. Lomustine is an approved treatment option for recurrent GBM and has also been frequently administered in clinical trials as the standard treatment (26).

In our study, lomustine plus bevacizumab showed a positive effect not only on PFS but also on OS in GBM patients. In addition, we found that the combination significantly improved 6-month PFS in GBM patients. These results are different to a previous meta-analysis (14) study which had explored the efficacy of lomustine plus bevacizumab in progressive GBM and which showed that treating patients with bevacizumab and lomustine could improve PFS significantly compared to control groups. However, there was no significant difference on OS. Because the previous study (14) did not provide a subgroup analysis, we performed some subgroup analyses based on the different control groups and drug doses to determine whether the results from the six studies included in our study, were comparable.

It is a remarkable fact that the dose selection we obtained through the subgroup analysis is different from previous study (27) results. The previous study (27) emphasized that the potential negative consequences of higher doses of bevacizumab are related to the promotion of tumor hypoxia, a well-known therapeutic tolerant medium, and the promotion of the aggressive phenotype of GBM. Furthermore, in a retrospective analysis, bevacizumab at low dose strength (<5 mg/kg/week) can improve PFS and OS better than bevacizumab at normal dose strength, and for patients with high-grade glioma, there was an inverse relationship between the dose intensity of bevacizumab and OS (*r* = 0.48, *p* > 0.00001) (28). It was hypothesized that lower doses of anti-angiogenic therapy may potentially improve the delivery of chemotherapeutic drugs and ultimately improve the prognosis of patients. However, in our study, the improvement in survival outcomes was more pronounced in Dose group 2 than in Dose group 1, which suggests that the bevacizumab dose may not be inversely related to OS. Moreover, based on the results of Dose group 3, we concluded that at the same dose of bevacizumab, an excessive dose of lomustine may be detrimental to OS. Although this study shows that Dose group 2 resulted in the best outcomes, the grouping is still not detailed enough. In future trials, more doses should be tested to determine the best dose combination, and the corresponding adverse reactions.

The mechanism behind the observed increase in OS, PFS, and 6-month PFS, when patients are treated with both lomustine and bevacizumab, is still not completely clear. It has been proposed that the normalization of vessels around the tumor, improvement of regional cerebral blood flow, augmentation of the antitumor effects of chemotherapy and radiotherapy are key components of the antiangiogenic activity (29–31), but more studies are needed.

Since there are not enough clinical studies on lomustine combined with bevacizumab in the treatment of GBM, only six studies were included. There may be some bias in these conclusions and a more systematic and theoretical analysis is required to determine the effectiveness of this drug combination in GBM. At present, the results of lomustine combined with bevacizumab are still encouraging for clinical treatment of GBM, and Dose group 2 is a potential option that provides a good starting point for discussions and further clinical trials.

## Data Availability Statement

The original contributions presented in the study are included in the article/Supplementary Material, further inquiries can be directed to the corresponding author/s.

## Author Contributions

XR: the main author of this article and the thought provider of this meta-analysis. DA: provided technical guidance for the meta-analysis. TL: assisting in writing articles, searching literature, and proofreading. LX: mainly responsible for the editing of figures and tables. LS: responsible for overall supervision. All authors contributed to the article and approved the submitted version.

## Conflict of Interest

The authors declare that the research was conducted in the absence of any commercial or financial relationships that could be construed as a potential conflict of interest.
