# Supplementary material for: Effectiveness of Lomustine Combined With Bevacizumab in Glioblastoma: A Meta-Analysis
Source: Front Neurol. 2021 Jan 20;11:603947. doi: 10.3389/fneur.2020.603947 (PMC7855027; doi:10.3389/fneur.2020.603947)
Supplement: Supplementary file 1 [file Table_1.DOCX]

| **Cochrane risk of bias for randomized studies** | Brandes 2019 | Taal 2014 a | Taal 2014 b | Weathers 2016 | Wick  2017 |
| --- | --- | --- | --- | --- | --- |
| 1. Random sequence generation (selection bias) | Unclear | Low | Low | Unclear | Unclear |
| 2. Allocation concealment (selection bias) | Low | Low | Low | High | Low |
| 3.Blinding of participants and personnel (performance bias) | Low | High | High | Low | High |
| 4. Blinding of outcome assessment (detection bias) | Low | High | High | Low | High |
| 5. Incomplele outcome data (attrition bias) | Low | Low | Low | Low | Low |
| 6. Selective reporting (reporting bias) | Low | Low | Low | Low | Low |
| 7. Other bias | Unclear | Low | Low | Unclear | Low |

**Supplementary Material**

**Supplementary Tables：**

**Supplementary Table 1：The quality assessment of the included non-randomized studies. The quality of the included literature was assessed using MINORS for nonrandomized trials.**

| **Methodological item for non-randomized studies** | Heiland 2016 | Jakobsen 2018 |
| --- | --- | --- |
| 1. A clearly stated aim | 2 | 2 |
| 2. Inclusion of consecutive patients | 2 | 2 |
| 3. Prospective collection of data | 2 | 2 |
| 4. Endpoints appropriate to the aim of the study | 2 | 2 |
| 5. Unbiased assessment of the study endpoint | 0 | 0 |
| 6. Follow-up period appropriate to the aim of the study | 1 | 0 |
| 7. Loss to follow up less than 5% | 1 | 1 |
| 8. Prospective calculation of the study size | 0 | 0 |
| Additional criteria in the case of comparative studies |  |  |
| 9. An adequate control group | 2 | 2 |
| 10. Contemporary groups | 2 | 1 |
| 11. Baseline equivalence of groups | 2 | 2 |
| 12. Adequate statistical analyses | 2 | 2 |

**Supplementary Table 2: Quality assessment of the included RCT studies. RCT, randomized controlled trial.**

**Search strategies**

**Source: PubMed**

**Searched on:** June 6, 2020

**Results:** 73

| **Search** | **Query** | **Results** |
| --- | --- | --- |
| #1 | "Glioblastoma"[MeSH] | 25,011 |
| #2 | "glioblastoma*"[Title/Abstract] OR "glioblastoma multiforme"[Title/Abstract] OR "giant cell glioblastoma"[Title/Abstract] OR "grade IV astrocytoma"[Title/Abstract] | 37,387 |
| #3 | #1 OR #2 | 42,206 |
| #4 | "Lomustine"[mesh] | 2,282 |
| #5 | "Lomustine"[Title/Abstract] or "CCNU"[Title/Abstract]) | 2,216 |
| #6 | #4 OR #5 | 3,145 |
| #7  #8  #9  #10 | "Bevacizumab"[MeSH]  "Bevacizumab"[Title/Abstract] OR "Mvasi"[Title/Abstract] OR "Avastin"[Title/Abstract])  #7 OR #8  #3 AND #6 AND #9 | 11,476  16,165  18,100  73 |
